#  Protecting children across borders – child protection in an international context (Germany/Switzerland) as an interprofessional teaching unit

**DOI:** 10.3205/zma001297

**Published:** 2020-02-17

**Authors:** Franziska Krampe, Stephanie Peters, Christine Straub, Sebastian Bode

**Affiliations:** 1 University of Freiburg, Center for Pediatrics, Department of general pediatrics, adolescent medicine, and neonatology, Medical Center, Freiburg im Breisgau, Germany

**Keywords:** interprofessionalism, internationality, course, pediatrics, child protection, IPE, IPC

## Abstract

**Introduction: **Interprofessional collaboration (IPC) in everyday clinical practice is a prerequisite for good patient care but currently this is not sufficiently anchored in the education of health care professionals.

**Project description:** A course on child protection in the interprofessional and international domain was established at the Medical School, University of Freiburg. In this course, students of medicine, nursing science and social work acquire skills for successful interprofessional cooperation. Its participants learn across professional and national borders, not only with but also from and about each other. In this way, they deepen their insights into international IPC through a key topic that is relevant to many disciplines. The course is run as a one-day campus day. This paper presents the course setup and evaluation results.

**Methods: **The evaluation was carried out online and in writing in a before and after format using the Freiburg Questionnaire for Interprofessional Learning Evaluation (FILE) in addition to oral feedback. Learning objectives for IPC and child protection were formulated and the participants were asked about their subjective achievements.

**Results:** From summer semester (SuSe) 2017 to SuSe 2018, 39 participants took part in the course. It was rated as m=1.5 (using German school grades where 1=very good, 6=unsatisfactory). In 18 of the 26 FILE items, participants report a self-assessed increase in knowledge or skills/abilities. This growth in learning coincides with the learning objectives set.

**Discussion & conclusion: **From the perspective of the participants, the course teaches interprofessional competencies in an international setting and is seen as an informative course offer. The continuation or expansion of such courses as a supplement to purely single-country interprofessional courses is desirable.

## Zusammenfassung

**Einleitung: **Interprofessionelle Zusammenarbeit (Interprofessional Collaboration, IPC) im klinischen Alltag ist Voraussetzung für eine gute PatientInnenversorgung, bisher bereitet die Ausbildung in Gesundheitsberufen darauf aber zu wenig vor.

**Projektbeschreibung: **In Freiburg i. Br. wurde eine Lehrveranstaltung (LV) zum Kinderschutz im interprofessionellen und internationalen Umfeld etabliert. Dort erwerben Studierende der Medizin, Pflegewissenschaft und Sozialen Arbeit gemeinsam Kompetenzen für eine erfolgreiche interprofessionelle (IP) Zusammenarbeit. Dabei lernen die Teilnehmenden (TN) grenzüberschreitend nicht nur mit-, sondern auch von- und übereinander. Sie vertiefen so den bedeutenden Aspekt der internationalen IPC beispielhaft an einem für viele Fachrichtungen relevanten Thema. Die LV findet als eintägiger Campustag statt. Diese Arbeit stellt den Ablauf der Lehrveranstaltung und Evaluationsergebnisse vor.

**Methoden: **Evaluiert wurde online und schriftlich in einem prä-post-Format mit dem Freiburg Questionnaire for Interprofessional Learning Evaluation (FILE), zudem erfolgt ein mündliches Feedback. Lernziele zur IPC und zum Kinderschutz wurden formuliert und die TN zu deren subjektiven Erreichen befragt.

**Ergebnisse: **Von Sommersemester (SoSe) 2017 bis SoSe 2018 nahmen 39 TN an der LV teil. Sie wurde mit m=1,5 (deutsche Schulnoten) bewertet. In 18/26 Items des FILE berichten die TN von einer selbsteingeschätzten Zunahme von Wissen oder Fertig-/Fähigkeiten. Dieser Lernzuwachs deckt sich mit den gesetzten Lernzielen.

**Diskussion & Schlussfolgerung: **Aus Sicht der TN vermittelt die LV IP Kompetenzen im internationalen Setting wird als lehrreiches Angebot wahrgenommen. Die Weiterführung bzw. ein Ausbau solcher LV als Ergänzung zu mononationalen IP LV ist wünschenswert.

## 1. Introduction

Interprofessional education (IPE) is the mutual learning from, with and about each other by learners from two or more professional groups to improve the cooperation between the professional groups and patient care [1]. Interprofessional learning (IPL) creates such interaction as a result of interprofessional education (IPE) or spontaneously in the workplace [2], [3], [4]. IPE can form the basis for successful interprofessional collaboration (IPC). IPC is essential for good patient-centered care in the health care system [5], [6], [7], [8], [9], [10], [11], [12]. However, medical studies or the curriculum of other health professions so far insufficiently prepare professionals for this type of collaboration [11], [13], [14]. 

The *National Competence-Based Catalog of Learning Objectives in Medicine* (NKLM) in Germany, like the *Swiss Catalog of Learning Objectives for Undergraduate Medical Training* in Switzerland, requires medical students to learn the competencies necessary for successful interprofessional collaboration [http://www.nklm.de], [15], [16]. Among other things, they are tasked to learn how to behave respectfully in interprofessional collaboration, to know their own competences and limits in an interprofessional team and to develop an understanding of their own role and the role of others [http://www.nklm.de]. Similar concepts like the NKLM also exist for medical students, for example in Great Britain, Canada or Scandinavia [17], [18], [19]. The Masterplan for Medical Studies 2020 [20] aims to improve the communication skills of medical students and to increase the number of jointly taught courses for students in health professions. There is also a call for training (pediatric) nurses (training and examination regulations) and social workers (core curriculum of the German Society for Social Work) to successfully work together in interprofessional teams [21], [https://www.buzer.de/gesetz/4330/a175620.htm]. It is not specified what form this interprofessional learning should take [22]. An integration of interprofessional courses into the curriculum is desirable to underline the importance of IPE for the quality of patient care and patient safety [23], [24], [25]. 

For children and adolescents, interprofessional collaboration is important beyond the medical setting. Cooperation between families, schools or kindergartens is necessary in order to promote the individual development of children and for providing adequate care [26], [27]. In pediatrics, pediatric surgery and all other disciplines dealing with the medical care of children and adolescents, child protection is an important example of the special importance of interprofessional collaboration, in which all professional groups (including doctors, nurses, social workers, educators, teachers and physiotherapists) must work together for the benefit of the child and its environment [28], [29], [30][. In a previous study by our group we were able to show that the topic of child protection is a suitable vehicle for imparting core interprofessional competences. The results of this single-country course showed, among other things, a self-assessed improvement in interprofessional skills and awareness of the importance of the topic [31]. This coincides with the results of other interprofessional courses, in which the participants indicated an increased awareness of the need for interprofessional cooperation after participating in an interprofessional course [32]. 

Due to the geographical location of Freiburg im Breisgau, it is often necessary to work across borders in everyday medical work. At the same time, the current curricula do not adequately prepare students for this aspect. The course* “Better cooperation, better help?!” Interprofessional child protection in pediatrics in the German / Swiss international context (KiPÄDiK – D/CH)* aims to prepare the participants for international interprofessional cooperation and to strengthen the cooperation between the participating universities and clinical care facilities in the border triangle. For this purpose, the course was included in the EUCOR (The European Campus) program, which promotes the cooperation of the universities on the Upper Rhine Valley in France, Germany and Switzerland [https://www.eucor-uni.org/de/]. The course setup and the results of the evaluation are described below.

## 2. Project description

### Framework conditions of the course

The course is designed as a one-day facultative campus day within the *Longitudinal Strand of Interprofessionality* (LongStI [https://www.medstudek.uni-freiburg.de/studienganguebergreifende-bereiche/kompetenzzentrum/bmbf-verbundprojekt-merlin/longitudinale-straenge/longitudinale-straenge]) at the Medical Faculty Freiburg and EUCOR – The European Campus and has been taking place once a semester in Freiburg im Breisgau since the summer semester (SuSe) 2017. Social work students of ESTES Strasbourg, medical students of the University of Strasbourg and nursing staff of the University Hospital of Strasbourg were invited from the French side; from the German side medical students of the Albert-Ludwigs University of Freiburg im Breisgau, social work students at the Protestant University and students of nursing science at the Catholic University; and from the Swiss side social work students at the University of Applied Sciences Northwestern Switzerland and students of nursing science at the University of Basel.

The interprofessional and international management and lecturing team consists of a paediatrician (D), a social scientist (D) and a health educator (D) from the Center for Pediatrics and Adolescent Medicine at the University Hospital Freiburg and is supplemented by various key note lectures on the subject by international lecturers from various disciplines (medicine (D), social work (D/F/CH), nursing science (D). The cooperation of different professional groups in a transnational context can thus be experienced directly by the students. The language of instruction is German. If necessary, simultaneous translation into French is available.

#### Learning objectives

The learning objectives (see table 1 (Tab. 1)) include understanding your own professional role as well as differentiating yourself from the professional role of others in an international context and recognizing the limits of your own actions. In addition, basic knowledge of the child protection system in Germany, France and Switzerland is to be acquired.

#### Conceptual framework

The present course is a further developed form of the single-country course* “Better cooperation, better help?!” – Interprofessional learning in pediatrics using the example of early intervention and child protection* [31]. The course is founded on the guidelines for interprofessional training and takes direction from the requirements of the Masterplan for Medical Studies 2020 as well as the training and examination regulations for nursing professions [1], [20], [33], [https://www.buzer.de/gesetz/4330/a175620.htm]. Elements of learning (group work, sufficient time for joint discussion, practice-oriented course content using realistic case vignettes) from the single-country course that proved reliable were retained, so that the course runs with only a modicum of frontal teaching (keynote speeches). The participants work together in groups, team building units and plenary discussions to acquire the most important learning content. In addition, information and teaching materials for self-study on interprofessional work, communication, and a guide for organizing a case conference for the preparation and follow-up of the course are provided as part of the event.

#### Course setup

**Figure 1:**
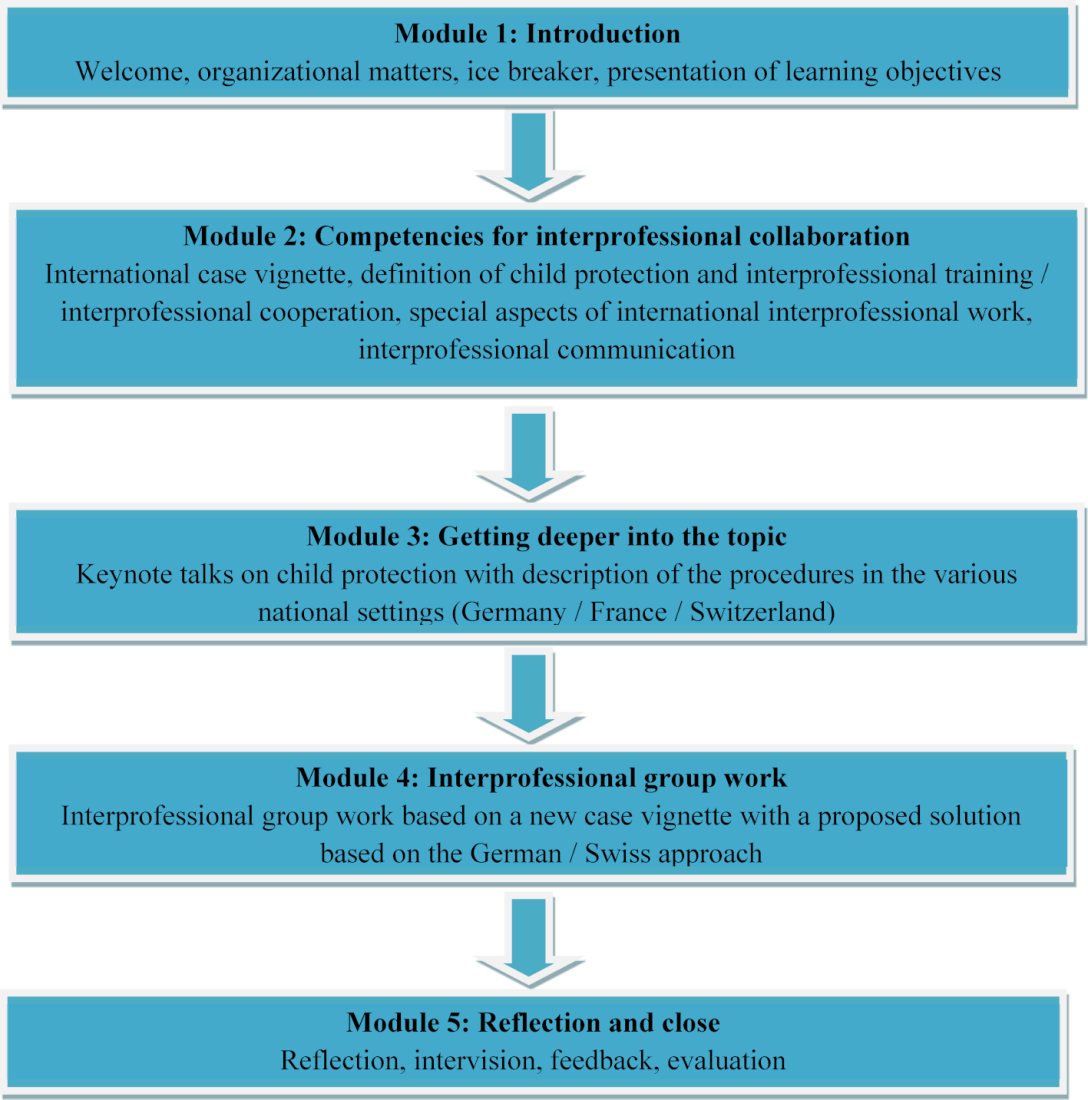
Course schedule

Figure 1 (Fig. 1) shows the course setup. The course design requires participants to work through five blocks in sequence.

1. **Introduction: **After an introduction, there is an ice breaker round in which the participants get to know each other better regarding their respective professional roles.
2. **Competences for interprofessional collaboration: **The participants get an insight into the topic of child protection using an exemplary case vignette. In addition, definitions of terminology relevant to IPE and IPC are discussed. Finally, the participants practice and consolidate the common (technical) language in a group task and practice successful communication strategies for interprofessional and international cooperation.
3. **Topic consolidation:** Short keynote talks on child protection in Germany, France and Switzerland serve to intensify engagement with the topic.
4. **Interprofessional group work: **The participants process a new case vignette in small groups and are given sufficient time for interprofessional and international exchange.
5. **Reflection and intervision: **The last block consists of reflection and intervision of the interprofessional group work, with the aim of identifying enabling and inhibiting factors for interprofessional and international cooperation.

## 3. Methods

The evaluation is carried out in a before and after format, first online and then in writing using the *Freiburg Questionnaire for Interprofessional Learning Evaluation* (FILE) [34]. FILE comprises 21 items, which the participants rate themselves on a five-point Likert scale ranging from “1=does not apply at all/do not agree at all” to “5=applies/agree completely”. FILE takes into account the dimensions of knowledge, teamwork, reflection, attitude and participation in optimal health care. In addition, the questionnaire contains a code for assigning the before and after questionnaire and demographic information. The questionnaire filled in after the course also includes five FILE items on interprofessional issues, five items on skills acquisition, five questions on the general assessment of the course and five open questions for free text comments. The five questions on the general assessment of the course include questions on the overall assessment of the course, assessment of the teaching team, interprofessional collaboration of the teaching team, importance of interprofessional course offers and continuation of the course. They were assessed by the participants using German school grades (1=very good, 6=unsatisfactory) or rated on a 5-point Likert scale. The statistical analysis was done using SPSS (version 23.0. and 25.0. Armonk, NY: IBM Corp.). The evaluation was carried out as descriptive statistic (absolute and relative frequencies, group comparisons using t-tests and simple analysis of variance). In addition, oral feedback was given at the end of the course. The free text responses in the questionnaire and the statements from the oral feedback were evaluated using quantitative text analysis.

## 4. Results

To date, n=39 participants were recruited for the course. 27 (69.2%) of the participants were female and 12 (30.8%) male. 12 medical students from Germany, 15 nursing students (CH n=3, D n=12) and 12 social work students (CH n=11, D n=1) took part. On average the participants were 30 years of age (21-58 years). 36 before and 39 after questionnaires were included in the evaluation. For organizational reasons, it was not possible to recruit participants from France. Both the course and the teaching team were rated as good (see table 2 (Tab. 2)). The item “The lecturers were good representatives of interprofessional collaboration” was rated m=4.65±0.54 (n=39) on a 5-point Likert scale (“1=does not apply at all” to “5=applies completely”). The evaluations did not differ significantly between the different professional groups (social work: m=4.64±0.51; human medicine: m=4.50±0.67; nursing science: m=4.79±0.43; p=n.s.). All participants advocated the continuation of the course.

### Interprofessional learning and relevance of the topic

Table 3 (Tab. 3) and table 4 (Tab. 4) show the improvement of the interprofessional competences as self-assessed by the participants as well as the items rated not significantly differently with regard to IPL. Table 5 (Tab. 5) shows the items regarding the relevance of the topic. No differences were found in the before and after comparison based on gender or the professional group.

#### Self-assessed skills and competence acquisition 

**Figure 2:**
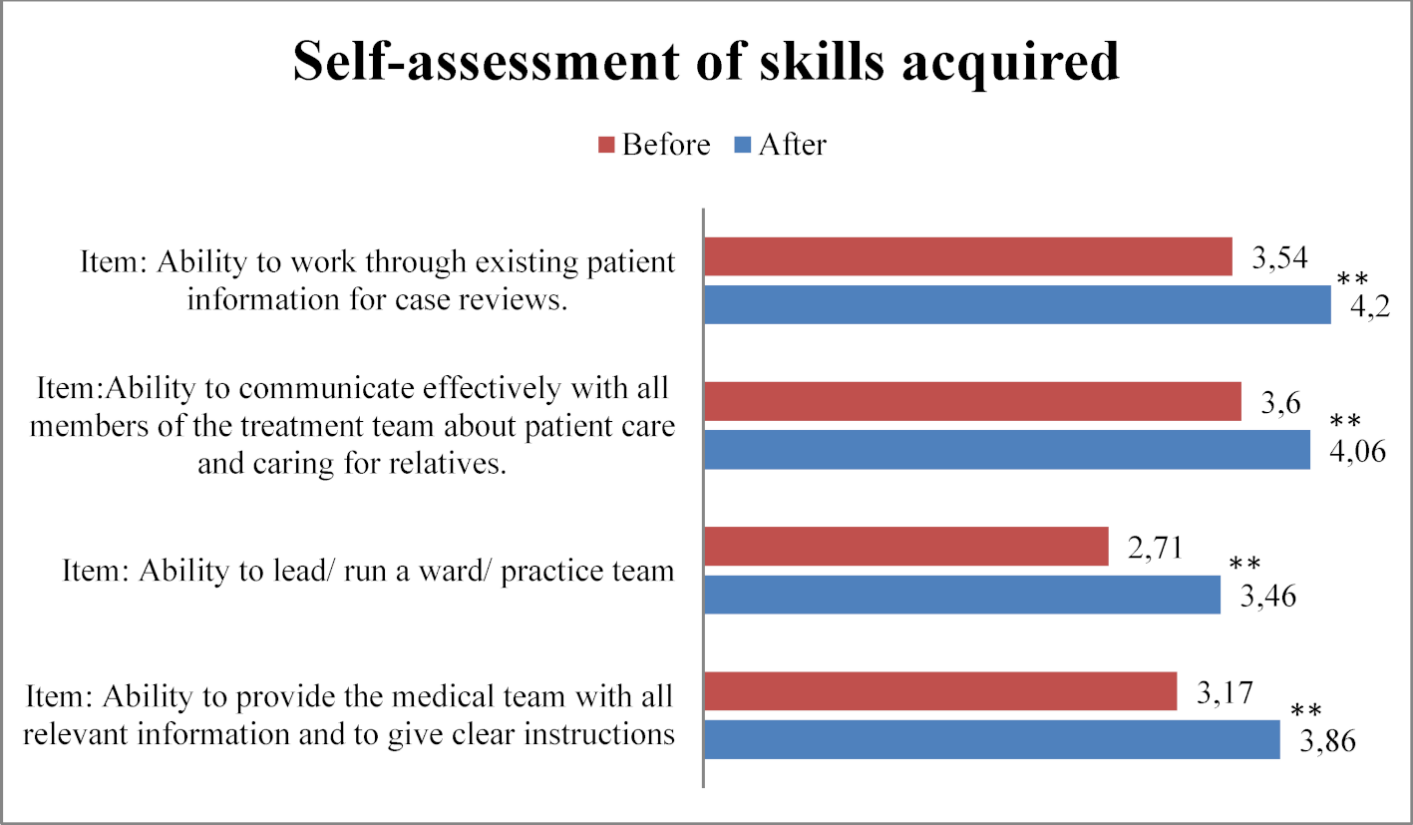
Self-estimated significant acquisition of skills in pre/post comparison. Possible answers on a five-level Likert scale (1=does not apply at all, 5=applies completely). Items 5.1, 5.2 and 5.4 refer to the learning objectives and are marked in italics. First, second and fourth item refer to the learning objectives and are marked in italics.

14 of 21 FILE items showed significantly higher self-assessed competence after the course. Table 6 (Tab. 6) shows the items with a significant difference in the before and after survey, nine of which depict the course learning objectives. A self-assessed increase for skill acquisition was shown by the participants in 4/5 items. Figure 2 (Fig. 2) shows the self-assessed skill acquisition of these four skills in the before and after comparison, three of which relate to the learning objectives. There was no significant difference in the assessment of the item “Ability to assess one’s own level of competence and possibly seek advice from others”; before with m=4.06±0.84 and afterwards with m=4.37±0.77 (p=n.s.).

#### Internationality

The participants reported on their experiences of the challenges in international cooperation in everyday work and emphasized the positive aspect of international exchange as part of their education in the free text comments and in the oral feedback (see table 7 (Tab. 7)).

#### Free text comments and oral feedback

The participants stressed their positive views of the course design (“planning and presentation of the content [was particularly good]”, “a lot of variety in the event”, “very good concept”). The participants rated internationality (n=15), better understanding of roles (n=14) and skills acquisition for effective conflict resolution (n=7) as positive (see table 6 (Tab. 6)). With regard to interprofessional collaboration, the participants gave 29 positive statements in the oral feedback and the free text responses. The participants stated that learning more about the work of social workers as an important learning result for them (n=12). Social work students said they had gained more confidence in their profession and found that they were a part of the team just as important as doctors or nurses (n=5). The participants overall wanted an even stronger focus on practical examples (n=8) and even more opportunities for group work (n=5) as well as making the course longer (n=3).

## 5. Discussion

The good evaluation of the course, the teaching team and the interprofessional cooperation of the teachers shows an overall good acceptance of the course by the participants. The participants particularly appreciated having sufficient time for interprofessional and international exchange in the course. Due to the optional nature of the course, the participants’ and lecturers’ motivation can be assumed to be high, which can help explain the positive overall assessment of the course. A high level of willingness for interprofessional cooperation or an awareness of the importance of IPE in this cohort is also visible in the positive rating of corresponding items in the before questionnaire. The item of self-assessed relevance of interprofessional course offerings, however, shows an increase following the course. The relevance of interprofessionalism in health care was also highly valued, with a significant increase in the before and after comparison. After the course, the participants assessed themselves as knowing more about the interprofessional care of patients and when they should seek advice from other professional groups. This coincides with results from the literature [32].

After the course, the participants stated that they critically questioned their actions in the interprofessional team, knew their personal limits and the responsibilities of their own professional group as well as those of the other professional groups and were able to communicate with colleagues from other professional groups in a comprehensible manner. The participants also made very positive comments regarding awareness of their own role. Other authors also concluded that participants in interprofessional courses were able to acquire a clearer view of their role and to recognize personal and professional limits [35], [36], [37], [38], [39]. One limitation that has to be mentioned is that some of the evaluations before the course were already positive, so that while suggestions of a further increase were evident, significant change could not be shown in all items. Nevertheless, a possible conclusion is that the learning objectives regarding understanding of roles, conflict resolution and understanding of the relevance of interprofessionalism were achieved based on the self-assessment of the participants. This coincides with the results already reported [31], [32], [40]. Overall, the present work underlines that an IPE course on child protection can also be carried out in an international format. This could constitute a meaningful further development to complement the established single-country course on child protection, for instance in border regions [31].

The course expands the existing course offering of the *LongStI* of the Medical Faculty of Freiburg [https://www.medstudek.uni-freiburg.de/studienganguebergreifende-bereiche/kompetenzzentrum/bmbf-verbundprojekt-merlin/longitudinale-straenge/longitudinale-straenge] by including a specific pediatric subject area and, to our knowledge, represents the only international interprofessional course for health care professions in Germany. It would be desirable if there were more such offerings, especially in border regions, with a view to future possibilities of cross-border collaboration. In the free text comments and the oral feedback, the participants repeatedly expressed their wish for offering the course more often and for it to be included in the compulsory curriculum. The item “In education, workplace training and further education, it is necessary for health professionals to learn together” was rated significantly higher by the participants after the course. It would appear that the students were able to learn of the importance of IPE through the course presented here. This is good news, as in reality IPE has not yet been sufficiently implemented and many interprofessional competences are only acquired in the workplace [11], [13], [14]. Interprofessional learning offers such as the project presented here may contribute to the long-term implementation of IPE as part of the compulsory curriculum in medical studies in Germany, as is already the case in Sweden, for example [39], [41], [42], [43]. Fortunately, there are increasing offers for IPE and IPL at the medical faculties in Germany [13], [44], [45], [46], [47], [48], [https://www.medstudek.uni-freiburg.de/studienganguebergreifende-bereiche/kompetenzzentrum/bmbf-verbundprojekt-merlin/longitudinale-straenge/longitudinale-straenge], [https://www.bosch-stiftung.de/sites/default/files/documents/2018-01/Kurzbeschreibung_HIPSTA.pdf], [https://www.bosch-stiftung.de/sites/default/files/documents/2018-03/Projektbeschreibung_dt.pdf], [https://www.bosch-stiftung.de/sites/default/files/documents/2018-01/IPAPAED_Freiburg_Kurzbeschreibung.pdf]. An interprofessional course offering for students could be used to train multipliers for interprofessional teaching in Germany in the medium term and to meet the requirements of the NKLM [http://www.nklm.de] and the Masterplan for Medical Studies 2020 [20] with regard to cooperation with other professional groups in the health care sector.

### Challenges and limitations

Cross-border communication and organization with multiple participating universities and contact persons turned out to be a lengthy process, especially when areas of responsibility changed and new cooperation partners had to be found. For organizational reasons (including different semester times) it has so far not been possible to attract French students to take part in the course. Other interprofessional courses have also reported on the challenges of organization [31], [48], [49]. Internationality was not an additional challenge for the participants, especially since there was no language barrier. The international aspect was rated particularly positively. A limitation of the present study is the low number of participants (n=39) and the fact that only medical students from one country participated. In addition, the reported learning success is based on the participants’ self-assessment rather than objective assessment. When evaluating the overall results of the course, it must also be taken into account that it is an optional course and that a distortion of the results by a cohort of particularly interested, motivated, open-minded students cannot be excluded [45]. Nevertheless, the course presented here can serve as a possible example of an international interprofessional course and underline the high clinical relevance of child protection.

## 6. Conclusion

The course presented here enables medical, social work and nursing science students from Germany, Switzerland and potentially France to learn together, about and from one another. Using the example of child protection, it imparts interprofessional skills in an international context. The before and after evaluation of the course shows that the participants rate the course well and that the course learning goals were achieved in the self-assessment of the participants. The organization of the international course was a challenge. Opportunities should be created to enable more students to attend the course.

## List of abbreviations

- Total = total evaluation
- HM = human medicine
- IP = interprofessional
- IPC = interprofessional collaboration
- IPE = interprofessional education
- IPL = interprofessional learning
- M = mean
- N = number of participants/answers
- NKLM = National Competence-Based Catalog of Learning Objectives in Medicine
- SD = standard deviation
- NSc = nursing science
- SocW = social work

## Acknowledgements

The authors would like to thank all participants, teachers and colleagues of the Dean’s Office of the Medical Faculty of Freiburg for their support. We would also like to thank our colleagues at the University of Freiburg, the Catholic University of Freiburg, the Protestant University of Freiburg, the University of Applied Sciences of Northwestern Switzerland and the University of Basel for their outstanding cooperation. 

## Competing interests

The authors declare that they have no conflicts of interest in connection with this article. The project is part of the “LongStI” of the University of Freiburg, which is part of the project “MERLIN – Teaching Research in the Baden-Wuerttemberg Network” of the Federal Ministry of Education and Research. The course was awarded the *EUCOR – The European Campus* label, which made it possible for students from Switzerland to be awarded travel expenses.

## Figures and Tables

**Table 1:**
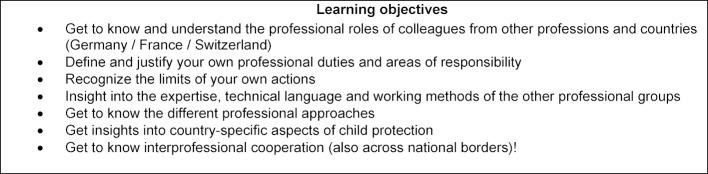
Learning objectives of the course

**Table 2:**
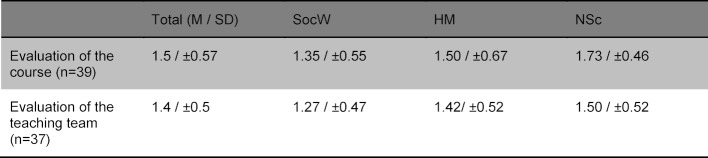
Evaluation of the course and the team of lecturers (rated on a Likert scale (1=very good, 6=unsatisfactory)).

**Table 3:**
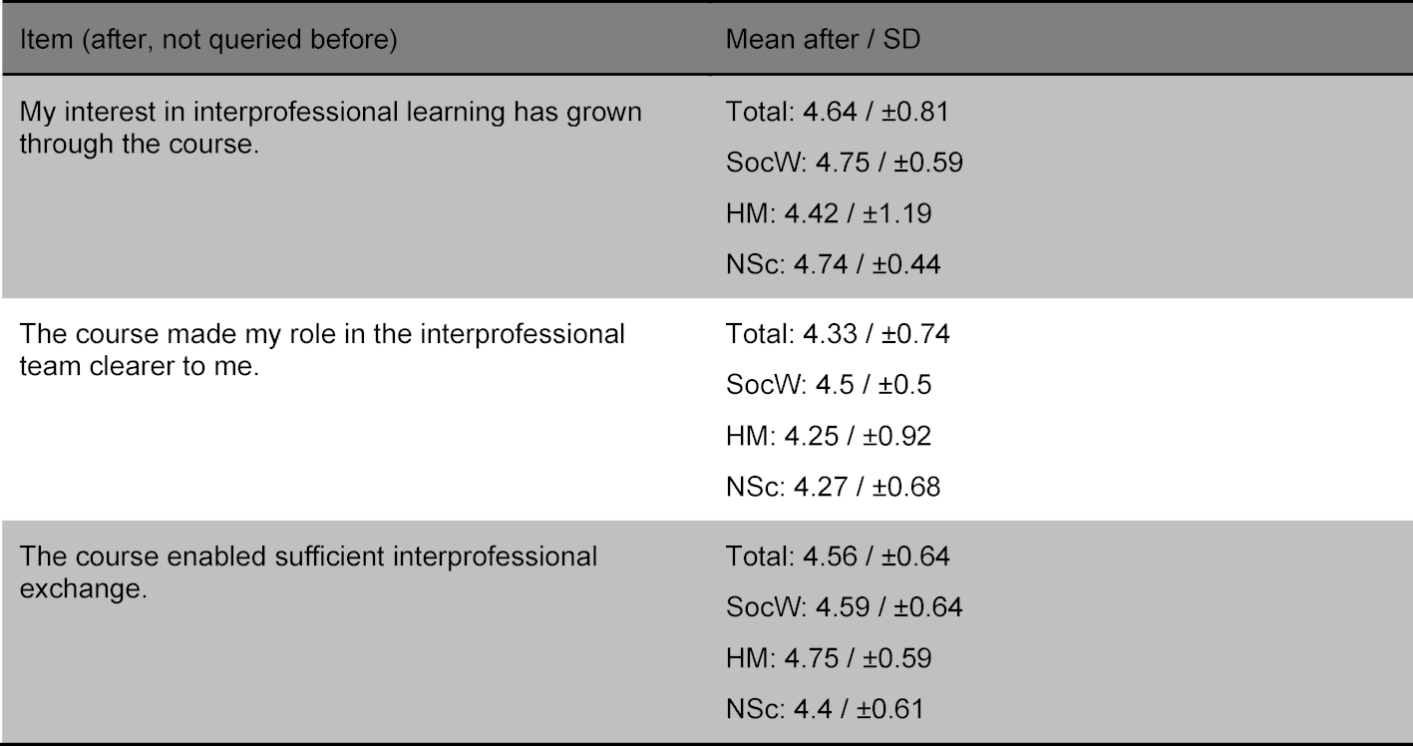
Self-evaluated improvement of competences regarding IPL by the participants (“1=does not apply at all/do not agree at all” to “5=applies/agree completely”).

**Table 4:**
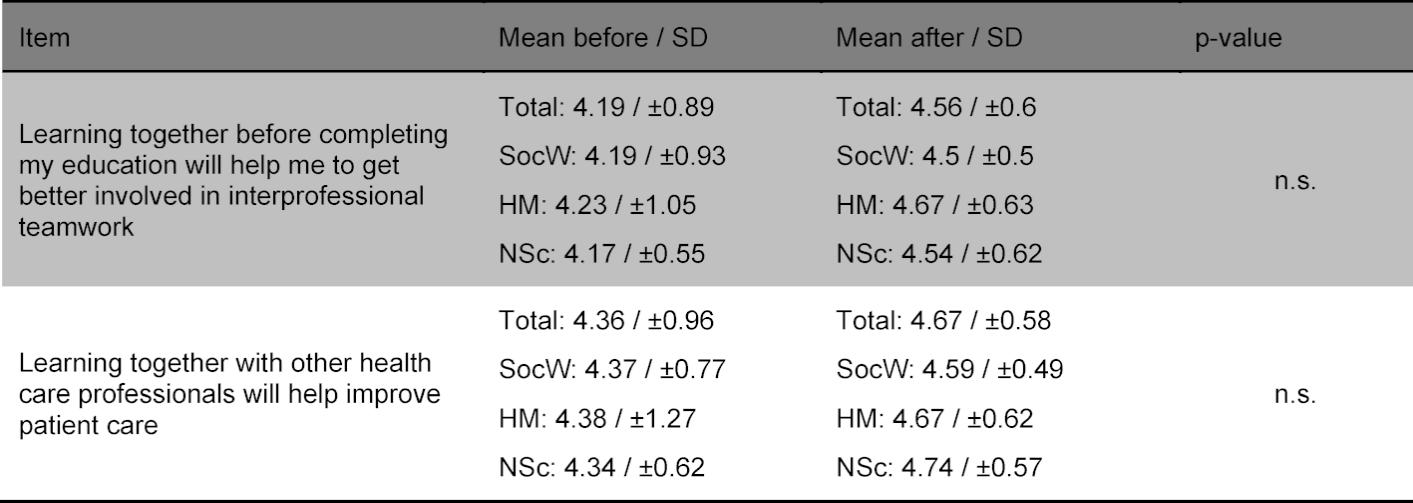
Non-significant items regarding interprofessional learning (“1=does not apply at all/do not agree at all” to “5=applies/agree completely”).

**Table 5:**
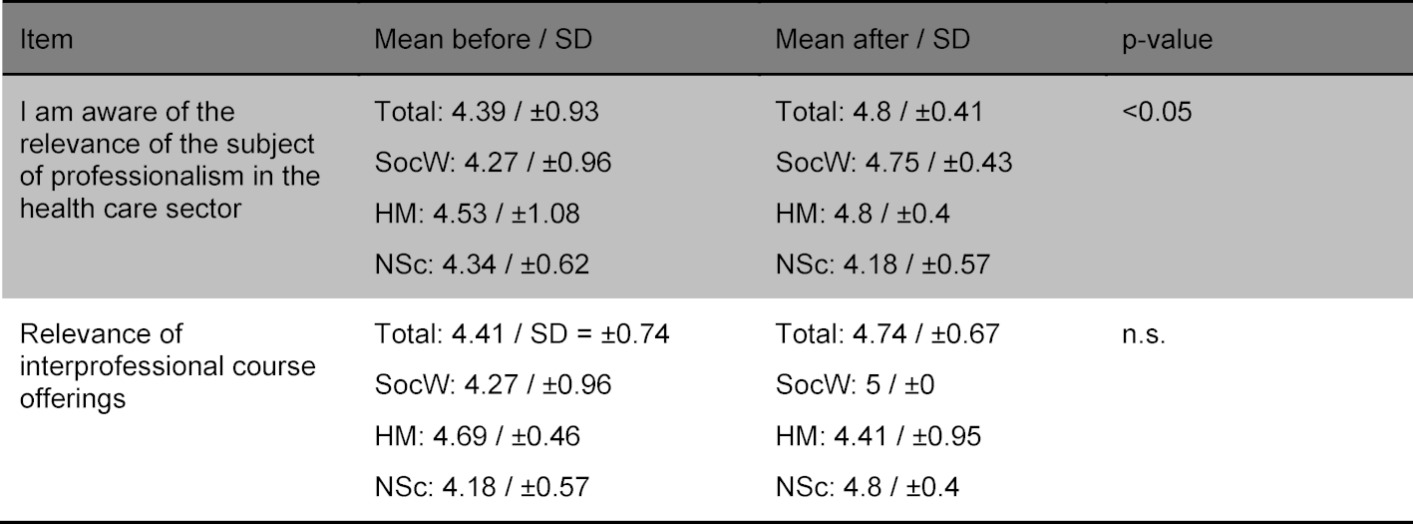
Items regarding the relevance of the topic “interprofessional collaboration” (“1=does not apply at all/do not agree at all” to “5=applies/agree completely”).

**Table 6:**
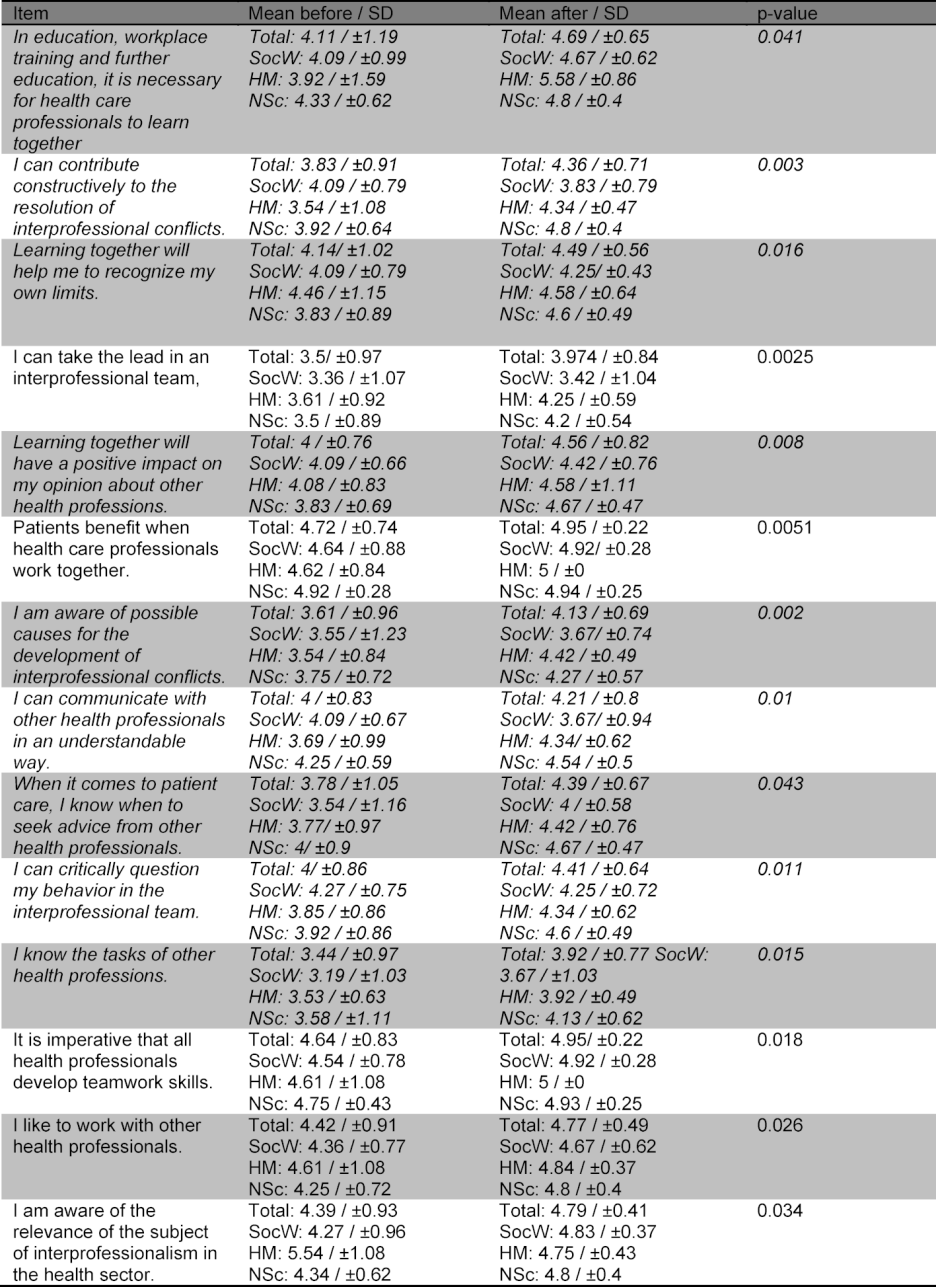
Significant items in the pre/post survey (1=does not apply at all, 5=applies completely). Items marked in italics refer to the learning objectives

**Table 7:**
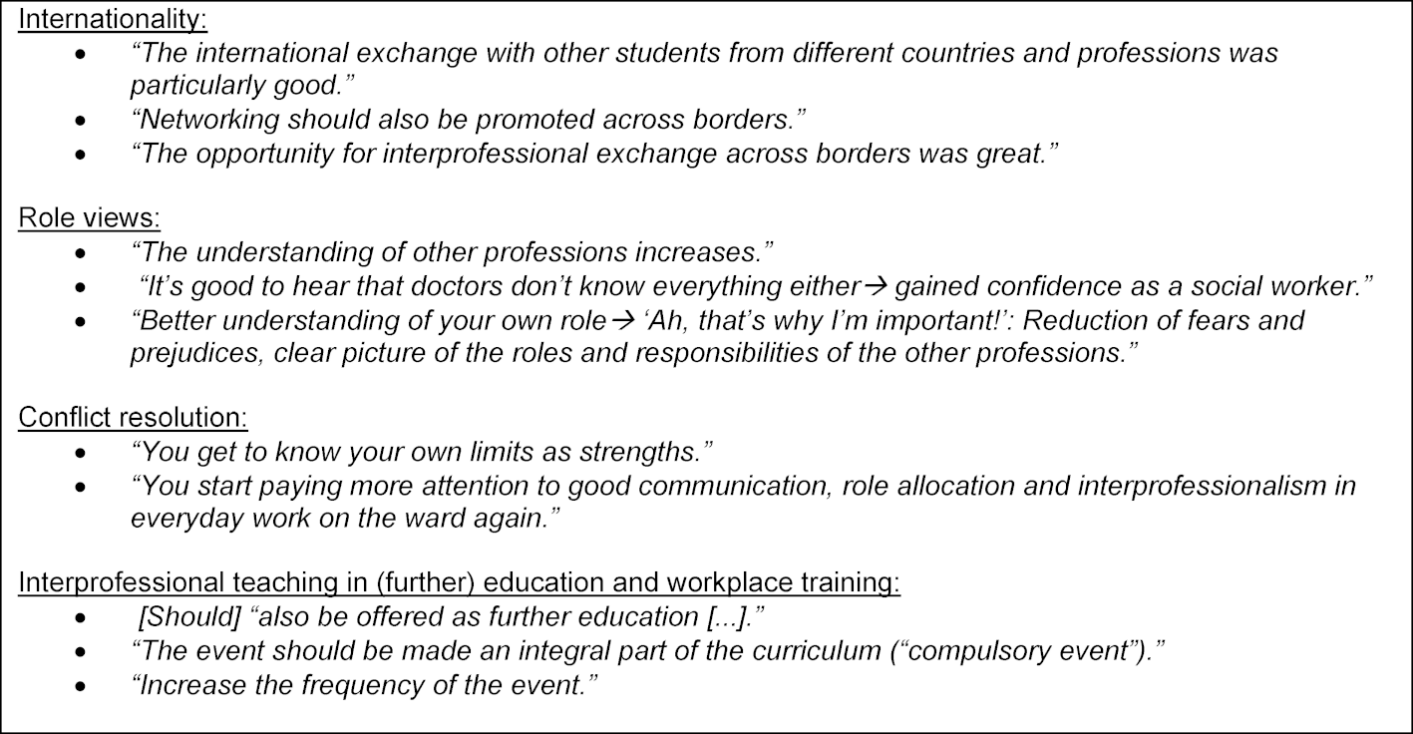
Quotations from the participants in the written evaluation and the oral feedback
